# Testing the feasibility of a knowledge translation intervention designed to improve chiropractic care for adults with neck pain disorders: study protocol for a pilot cluster-randomized controlled trial

**DOI:** 10.1186/s40814-016-0076-9

**Published:** 2016-07-20

**Authors:** Prakash Dhopte, Sara Ahmed, Nancy Mayo, Simon French, Jeffrey A. Quon, André Bussières

**Affiliations:** 1 School of Physical and Occupational Therapy, Faculty of Medicine, McGill University, Montréal, QC Canada; 2 Centre de Recherche Interdisciplinaire en Réadaptation (CRIR), Montréal, QC Canada; 3 Clinical Epidemiology, McGill University Health Center, Montréal, QC Canada; 4 School of Rehabilitation Therapy, Faculty of Health Sciences, Queen’s University, Kingston, ON Canada; 5 School of Population and Public Health, Faculty of Medicine, University of British Columbia, Vancouver, BC Canada; 6 International Collaboration on Repair Discoveries (ICORD), Vancouver Coastal Health Research Institute, Vancouver, BC Canada; 7 Spine Program, Department of Orthopaedics, Faculty of Medicine, University of British Columbia, Vancouver, BC Canada; 8 The Cambie Chiropractic Centre, Vancouver, BC Canada; 9 Département chiropratique, Université du Québec à Trois-Rivières, Trois-Rivières, QC Canada

**Keywords:** Neck pain, Knowledge translation, Chiropractors, Clinical practice guidelines, Multimodal care, Brief Action Planning, Feasibility outcomes, Cluster-randomized controlled trial

## Abstract

**Background:**

Neck pain in adults is common and a leading cause of physical disability. Recently, a guideline was developed for the management of non-specific neck pain (NSNP) with an aim to improve the quality of the delivery of chiropractic care. One key guideline recommendation is to undertake multimodal care for patients with NSNP. The aim of this pilot study is to determine the feasibility of implementing a multifaceted knowledge translation intervention by promoting the use of multimodal care by chiropractors managing patients with NSNP.

**Methods/design:**

The design is a cluster-randomized controlled pilot and feasibility trial. Chiropractors in private practice in Canada will be approached to participate in the study. Thirty consenting chiropractors will be randomized to receive either a theory-based educational intervention in the experimental group or simply a printed copy of the guideline in the control group. Each chiropractor will recruit five neck pain patients (a total of 150 patients) into the study. Development of the multifaceted intervention was informed by the results of a related qualitative study based on the Theoretical Domains Framework and consists of a series of three webinars, two online case scenarios, a self-management video on Brief Action Planning, and a printed copy of the practice guideline. *Primary feasibility outcomes* for both chiropractors and patients include rates of (1) recruitment, (2) retention, and (3) adherence to the intervention. A checklist of proxy measures embedded within patient encounter forms will be used to assess chiropractors’ compliance with guideline recommendations (e.g. exercise and self-care prescriptions) at study onset and at 3 months. Secondary outcomes include scores of behavioural constructs (level of knowledge and self-efficacy) for recommended multimodal care. *Clinical outcomes* include pain intensity and neck pain-specific disability. Analyses from this study will focus on generating point estimates and corresponding 95 % confidence intervals for parameters of a priori interest (recruitment, retention, adherence, pain intensity, Neck Disability Index).

**Discussion:**

Results of this study will inform the design of a larger cluster-randomized controlled trial aimed at evaluating the effectiveness of the theory-based tailored intervention and increasing the use of multimodal care by chiropractors managing patients with NSNP.

**Trial registration:**

https://clinicaltrials.gov/, NCT02483091

**Electronic supplementary material:**

The online version of this article (doi:10.1186/s40814-016-0076-9) contains supplementary material, which is available to authorized users.

## Background

Translating evidence into clinical practice is challenging. As a result, patients often fail to receive optimal care and may be exposed to unnecessary harm [1]. The Medical Research Council (MRC) guidelines on complex intervention evaluation recommends conducting feasibility and/or pilot studies with an aim to improve the effectiveness and efficiency of interventions and to address the challenges in translating research into real-world settings [2, 3]. One example where these recommendations apply is within clinical sites that deliver interventions for individuals with musculoskeletal (MSK) conditions.

Neck pain results in an enormous social, psychological, economic burden to society and is a leading cause of physical disability [4]. The estimated annual incidence of neck pain ranges between 10.4 and 21.3 % with a higher incidence noted in office and computer workers [5]. In chiropractic practice, neck pain accounts for approximately 25 % of initial consultations [6]. Opinions vary widely on what causes neck pain and how best to manage it [7]. The vast majority of patients with neck pain have symptoms that are “non-specific” in nature that cannot be attributed to a specific disease process or anatomical structure [8]. Perhaps as a result, relatively few treatments have been shown to achieve meaningful and sustained improvements in pain, physical function, and disability, despite associated high costs of neck pain [9].

Current evidence suggests a multimodal approach including manual therapy, providing self-management support to patients, and physical activity including exercise as an effective treatment strategy for acute and chronic neck pain [10]. The promotion of physical activity, including exercise, is a first-line treatment considered important in the prevention and treatment of musculoskeletal pain and its related co-morbidities [11]. For a minority of patients, clinician-delivered interventions and pharmacological treatments are appropriate, and in fewer cases, multidisciplinary pain management and/or surgery may be indicated [12]. In addition, multi- and/or inter-disciplinary multimodal therapy, as well as cross-sectorial integrated medical care appear to be cost-effective strategies for managing chronic pain [13].

Notwithstanding these recommendations, the contemporary management of non-specific neck pain (NSNP) is often suboptimal. For instance, a recent survey of Canadian chiropractors suggests that only 41 % of 2500 respondents provided advice to patients on self-management strategies. Another survey of chronic neck and back pain patients indicated that less than half of attending physicians, chiropractors, and physical therapists prescribed exercises [14]. Uninformative diagnostic testing, narcotics, and modalities tend to be over-utilized, while therapeutic exercise and activation tend to be under-utilized [13, 15]. For people with chronic NSNP, therapeutic exercise has a positive effect on pain and disability in the short (<1 month) and intermediate (1–6 months) terms [16]. However, when home exercises for neck or low back pain are prescribed, patient compliance is often poor with published adherence rates converging at about 50 % [17–19].

People with musculoskeletal pain will easily adopt an inactive lifestyle, possibly as a consequence of their physical impairment or because they believe that pain justifies physical inactivity [20]. Unfortunately, physical inactivity has major health effects worldwide. Physical inactivity is associated with many adverse health effects, including increased risks of coronary heart disease, type 2 diabetes, breast and colon cancers, and shorter life expectancy in general [21]. Ultimately, not only is increased compliance with prescribed condition-specific exercise likely to improve MSK-related complaints, but increased general physical activity may significantly reduce patients’ risks of developing serious co-morbidity.

A recent systematic review concluded that multifaceted knowledge translation (KT) interventions were no more effective than single-component KT interventions [22]. Nonetheless, there is growing evidence that active, multicomponent strategies are more effective in implementing change in professional behaviour [23–25]. Active, multicomponent strategies include the use of modalities such as interactive education [26] and printed educational materials (e.g. guidelines) [27]. In addition, a recent Cochrane review concluded that a tailored implementation intervention is more likely to improve professional practice than no intervention or dissemination of guidelines [28]. To date, very few studies have evaluated the impact of KT interventions in the chiropractic setting specifically [29].

### Context and purpose of the study

Recently, a clinical practice guideline (CPG) on the management of NSNP was updated with the aim of improving the quality of delivery of chiropractic care [30]. One of the key recommendations involves undertaking multimodal care for patients with acute and chronic NSNP. To facilitate the uptake of these recommendations, the Canadian Chiropractic Guideline Initiative (CCGI) (www.chiroguidelines.org) has developed and disseminated a multifaceted KT intervention (involving a combination of a webinar series, online clinical vignettes, and a learning module on self-care) [31]. Thus, the proposed overall aim of this study is to inform the design of a cluster-randomized controlled trial on the feasibility of implementing multimodal care in chiropractic practice.

### Research question and objectives

The research question of interest for a larger main study is the following: Among chiropractors in Canada, to what extent does the effectiveness of a multifaceted theory-based complex educational KT intervention plus provision of a copy of the CPG (for the intervention group) enhance behavioural change and compliance with a multimodal care programme when compared to the distribution of CPGs alone (for the control group) for the management of NSNP-related pain and disability over 3 months?

The immediate primary objective of the current pilot study is to provide evidence for the feasibility of conducting a fully powered cluster RCT to evaluate a complex KT intervention. Feasibility will be evaluated in terms of rates of recruitment, retention, and adherence to the study protocol. We will ascertain how closely participating chiropractors and patients adhere to the study protocol and will solicit feedback from them about the overall usefulness of the content and format of the KT intervention. The potential effectiveness of the complex KT intervention will also be estimated. Again, the results of this pilot trial will be used to design a full-scale cluster-randomized trial.

### Specific objectives

The specific objectives target two groups of participants: chiropractors and patients. For each group, both feasibility and efficacy potential will be estimated.

1. For chiropractors, the feasibility objectives are to estimate the proportion who
  - Are eligible to participate and are willing to be randomized;
  - Comply to all study procedures, including completing the KT intervention component and implementing the CPG recommendations; and
  - Complete the 3-month follow-up evaluation.
2. For patients, the feasibility objectives are to estimate the proportion who
  - Are eligible to participate and are willing to be randomized;
  - Adhere to all study procedures; and
  - Complete the 3-month follow-up visit and all questionnaires.
3. For chiropractors, the efficacy potential objectives are to estimate
  - The extent to which knowledge and self-efficacy changes after engaging in the KT intervention and CPG and
  - The extent to which knowledge and self-efficacy changes after engaging in CPG.
4. For patients, the efficacy potential objective is
  - To estimate the effect of their chiropractor’s KT intervention and CPG implementation changes in pain, disability, and satisfaction with care at the initial phase, as well as after 3 months of follow-up.
5. Other secondary objectives are to identify and provide solutions to
  - Chiropractors’ concerns about the quality of the webinars;
  - Potential impediments to successful initiation of the main study protocol after randomization; and
  - Challenges that participating clinicians have with managing the study (e.g. with implementing the multimodal care and/or completing initial and follow-up questionnaires).

## Methods/design

### Design

This is a pilot cluster-randomized, two-arm, parallel-group controlled trial with a 1:1 allocation ratio. A cluster-randomized design has been chosen to avoid contamination between intervention and control arms by individual patients who would potentially be served by the same chiropractor in a non-cluster design. In addition, cluster-randomized trials offer logistical convenience when implementing certain interventions such as training, feedback, and supervision programmes, which are easier to administer to groups rather than individuals [32].

The study will test the feasibility and impact on protocol adherence and patient outcomes of two methods of delivering an educational intervention: (1) a KT complex intervention (theory-based KT intervention that includes three webinars, two case scenarios followed by a quiz, and Brief Action Planning) plus dissemination of practice guidelines for the intervention group and (2) passive dissemination of a practice guideline alone for the control group.

### Study setting and location

Our study setting and location are private practices of licensed chiropractors in Canada.

### Subjects/population

#### Recruitment of chiropractors

A sampling frame of 8200 chiropractic practices within 10 provinces in Canada will be obtained from the Canadian Chiropractic Association (CCA). From this, a random sample of 200 chiropractors will be selected and approached for participation in this study [33, 34]. A sample of 200 chiropractors has been chosen as we expect that 20 % of eligible chiropractors will agree to participate (i.e. recruitment rate), and from these, 80 % will complete the study at 3 months (retention rate). Chiropractors who agree to participate and meet the eligibility criteria will be randomized. If we are unable to recruit the required sample of 30 from the first wave of 200 chiropractors, an additional 200 chiropractors will be randomly sampled.

##### Inclusion criteria

- Current registration with a provincial licensing boards and in active private practice in Canada;
- Graduation at least 1 year ago;
- Provision of chiropractic treatment to a minimum of two adults (age 18–65) with neck pain per week;
- Fluent in spoken English or French; and
- Access to the internet.

##### Exclusion criteria

Chiropractors will be excluded if they have already attended the webinar series or the self-management learning module. To date, over 700 Canadian chiropractors have registered for both the webinars and the self-management learning module, of whom over 475 have either completed it or are in progress of doing so. Prior registration provides the mechanism for confirming study ineligibility.

#### Recruitment of patients

Participating chiropractors will each recruit up to five consecutive new patients with neck pain. A recruitment/advertisement notice will be posted in each participating chiropractor’s waiting room. The expected recruitment of 5 patients within 3 months is reasonable assuming an average practice volume of 85 patient visits per week per chiropractor, of whom 25 % are expected to have neck pain [6].

##### Inclusion criteria

- Aged between 18 and 65 years, with a primary complaint of acute (<3 months) or chronic (>3 months) neck pain presenting as a new condition for treatment at the participating clinic;
- A diagnosis of NSNP (of any duration);
- Able to understand and speak English to complete all study questionnaires (which will be assessed by a designated member of the chiropractor’s office personnel at the time of screening).

##### Exclusion criteria

- Previous neck surgery;
- Presence of “red flags” (alerting the possibility of serious conditions such as malignancy, infection, fracture, inflammatory arthropathies including rheumatoid arthritis or vascular disease of the neck);
- Pregnancy; and
- Chiropractic care received in the preceding 3 months for a complaint of neck pain.

### Measures

The key feasibility outcomes of interest include (1) study recruitment/participation, (2) adherence to the intervention, (3) study retention, and (4) KT intervention effectiveness potential. Table 1 provides the different criteria for defining the outcomes derived from items (1) to (4) above. Table 2 further summarizes the feasibility outcomes, sources of measurement, and timing of administration.

**Table 1:** Criteria to assess feasibility

Construct	Parameter
	Eligibility proportion
Chiropractors	
Recruitment	Trial acceptance rate: >20 % agree to participate within 4 weeks
	Target population = 40 (assuming an 80 % retention rate)
Adherence to protocol	>90 % of participants will complete all 3 webinars, associated quizzes, 2 clinical vignettes, and a self-management learning module
Retention	80 % of participants will complete 3 months of patient follow-up
Patients	
Recruitment	Trial acceptance rate: 5 patients within 6 weeks of recruitment notice
	Target population = 150
Adherence to protocol	95 % will attend regular treatment sessions twice/week
	>80 % will comply with prescribed home exercise and physical activity
Retention	>80 % will complete patient encounter forms (VAS, NDI, and PSQ-18) and follow-up at 3 months

**Table 2:** Outcome measures

Outcome	Source	Description of measures	Data collection time points	
Feasibility				
Recruitment	Chiropractors and patients	Measured as a proportion of chiropractors and patients potentially eligible for participating	Initial stage	
		Eligibility rate = number of eligible chiropractors and patients divided by the number of invited chiropractors or patients		
		Participation rate = number of chiropractors and patients agreeing to participate divided by number of eligible chiropractors and patients		
Adherence to protocol	Chiropractors	For those randomized to intervention arm, measured through the rates of attendance of all 3 webinars, associated quizzes, completion of 2 clinical vignettes, and the self-management learning module	Within 6 weeks of assignment	
	Patients	Rate of adherence to follow-up visits, prescribed home exercise, and physical activity	Baseline	3 months
Adherence perception (knowledge and self-efficacy)	Chiropractors and patients	Completion of questionnaires	Baseline	3 months
Retention	Chiropractors and patients	Retention rate = number of chiropractors or patients who completed follow-up of all outcome measures at 3 months divided by number of chiropractors or patients who were randomized	Baseline	3 months
	Chiropractors	Rate of completion of patient encounter forms and questionnaires including levels of knowledge and self-efficacy and the BAP	Baseline	3 months
	Patients	Rate of completion of patient encounter forms and questionnaires including the BAP, visual analogue scale (VAS), Neck Disability Index (NDI), and satisfaction with care	Baseline	3 months

Differential retention and adherence rates across the randomized groups will also be measured

Expected adherence to multimodal care by chiropractors will be measured at the end of the study using a short self-administered questionnaire that will include items about knowledge (e.g. “I am following the recommendations regarding the use of clinical practice guidelines and multimodal approach for neck pain”) and self-efficacy (e.g. (i) “I am confident that I will not encounter difficulties in delivering multimodal care” and (ii) “If I encounter difficulties, I am confident that I can still offer multimodal care”) [29, 35, 36]. End-of-study questionnaires will also include the Brief Action Planning (BAP) skill survey and clinician experience in using the BAP tool in practice (Additional file 1).

Expected adherence to multimodal care by patients will also be assessed at baseline using a specific questionnaire item (e.g. “I intend to perform the neck exercises that were prescribed for my condition”). Furthermore, compliance with guideline recommendations at the patient level will be measured using a checklist documenting the extent of their confidence in managing most of their health problems, the type of help received from their treating chiropractor, and the use of exercise and self-care prescriptions, as well as their levels of compliance with recommended exercises (Additional file 1). Furthermore, patient-related health outcomes will be collected at baseline and at 3 months through the use of questionnaires to measure symptoms, impairment, activity interference at home and at work, general quality of life, and satisfaction with care (PSQ-18). The minimal clinically important difference (MCID) in the 10-cm VAS pain score will be measured as the mean difference between current and baseline scores among patient participants who report feeling either “a little worse” or “a little better” in terms of their global self-perceived change. Otherwise, a 10 % change from baseline will be considered minimal change, while a 30 % change from baseline will be considered a substantial and clinically important change [37].

The Neck Disability Index (NDI) is a 10-item self-administered questionnaire, scored from 0 to 50 with a higher score representing more disability. A score of 0 to 4 represents no disability, a score >35 represents complete disability, and a score >25 represents severe disability. An absolute change of 10 points or relative change of 20 % on the NDI will be considered clinically important [38].

Feasibility to conduct a larger study will also be determined by estimating the effect size (and subsequently, the anticipated sample size) needed for a future main trial. Challenges encountered by participating clinicians while conducting the trial will be assessed by administering an end-of-study questionnaire and conducting interviews. An open-ended question will be used to elicit the chiropractors’ experiences (challenges or facilitators) encountered while trying to comply with the guideline recommendations.

### Interventions

#### Development of the KT intervention

The science of KT research draws from a variety of behavioural and social science disciplines and employs new approaches and methods [39]. The proposed KT educational intervention was developed to facilitate the uptake of a recently developed guideline for the management of NSNP among chiropractors, the full details of which is published elsewhere [31]. To design the KT intervention, an expert panel used a systematic, theory-informed approach that was guided by the following key questions [40]:

1. Who needs to do what, differently?
  Based on wording from the neck pain guideline itself, the target-specified behaviour is the chiropractors’ adherence to recommended care, i.e. Undertaking or recommending multimodal care for patients with acute and chronic NSNP.
2. Using a theoretical framework, which barriers and enablers need to be addressed?
  Twenty-five chiropractors were invited to take part in telephone interviews guided by the Theoretical Domains Framework (TDF) [41] to specify modifiable barriers and facilitators to managing neck pain. The first 13 respondents from six Canadian provinces completed a 60-min interview. Transcripts were coded deductively by two independent assessors and reviewed by investigators. The results highlighted a number of potential barriers and facilitators to implementing a newly developed neck pain guideline targeting this professional group. Specifically, adherence to prescribing multimodal care was felt to be potentially influenced by nine key theoretical domains: (1) social influence; (2) environmental context and ressources; (3) reinforcement; (4) skills; (5) behavioural regulation; (6) knowledge; (7) memory, attention, and decision making processes; (8) social/professional role and identity; and (9) beliefs about consequences.
3. Which intervention components could overcome the modifiable barriers and enhance the enablers?
  An expert panel mapped behaviour change techniques to barriers and enablers within key theoretical domains and identified relevant KT strategies and modes of delivery to increase the use of multimodal care among chiropractors [42].

#### Components of the intervention

Table 3 describes the components of the intervention package. Four key elements were designed to capture key theoretical domains, behaviour change techniques, and modes of delivery. The specific learning objectives of the intervention components are presented in Additional file 2.

**Table 3:** Intervention components and modes of delivery

Key elements and topics	Delivery
(1) Three 50–60-min webinars containing didactic information on the following topics	
^a^Webinar 1. ^*^ Overview of what evidence-informed practice is and why CPGs are useful	CMCC continuing education (online)
Webinar 2. ^*^ Key recommendation of the new guideline on the management of non-specific neck pain	CMCC continuing education (online)
Webinar 3. ^*^ Introduction to self-management strategies and to the Brief Action Planning (BAP) model in particular	CMCC continuing education (online)
(2) Two online case scenarios each with care options to help apply recommendations as a proxy for daily practice with quizzes	Accessible on Fluid Survey after completion of the webinar 2 (neck pain guideline) at
	http://fluidsurveys.com/s/ClinicalVignette1 http://fluidsurveys.com/s/ClinicalVignette2
(3) A self-management video underpinned by the BAP model to demonstrate how clinicians can facilitate patient decisions about self-management strategies. The video portrays a clinician discussing active planning strategies with a chronic neck pain patient who chooses to increase his/her level of physical activity	Accessible online after completion of the webinar 3 (BAP) on the LMS of the CMCC through a link from the CCGI website at http://www.chiropractic.ca/guidelines-best-practice/Chiropractors/resources/physical-activity-ergonomics-public-health/

^a^Before watching webinar 1 on EIP, clinicians will be encouraged to complete three online modules (Evidence Informed Practice, Summary Research, and Assessing Summary Research) at http://www.csh.umn.edu/evidenceinformedpracticemodules/index.htm

*All three webinars were recorded between October 29 and November 26, 2014, for future diffusion to participants in the intervention group

#### Delivery of the intervention

Chiropractors consenting to participate will be allocated to receive either the KT strategies plus practice guidelines for the intervention group or practice guidelines alone for the control group. Modes of delivery of the webinars are outlined in Table 3. All webinars have been recorded and will be made available to participants allocated to the intervention arm.

#### Acceptability of the intervention to participants

Acceptability will not be assessed directly. However, adherence to multimodal care and initial rates of willingness to participate will be used as proxy measures of acceptability of the intervention to chiropractors and patients. It may be that the intervention and the trial processes will be acceptable to some participants (those who participated and adhered to the protocols) but not to others (those who chose not to take part, perhaps due to being put off by the intervention or the associated trial processes or by competing commitments). Post-randomization withdrawals in the control group may suggest that control participants are dissatisfied with their allocation. Reasons for not participating will be documented using a pre-defined checklist.

### Procedures

The CCA and provincial chiropractic associations will be asked to promote the study via their newsletters and by emails to chiropractors informing their members of the study purpose and encouraging them to participate in this study. An invitation letter with the McGill University letterhead, with a consent form, a demographic questionnaire, and a prepaid stamped and self-addressed envelope, will be sent to the sample of 200 chiropractors. Chiropractors expressing an interest in participating will receive a follow-up letter written in a standardized format, giving information about the study project. A follow-up invitation and a reminder to participate will be sent if initially an insufficient number of clinicians are enrolled. As an incentive to participate, chiropractors who complete all aspects of the study will be entered into a draw to win one of four $250 gift cards as a token of appreciation. In addition, most provincial chiropractic regulatory boards have pre-approved the KT intervention for 4 h of Continuing Education (CE). Certificates of completion are produced once the KT intervention is completed by chiropractors and all quizzes have been successfully answered. Obtaining a credit for the course may contribute to participant confidence regarding the experimental KT intervention and may improve study adherence.

In order to determine the eligibility of the participating chiropractors, a demographic questionnaire will be sent with the invitation package to inquire about their age, sex, years in practice, practice location (rural versus urban), chiropractic school attended, type of practice (solo versus multidisciplinary clinic), main chiropractic techniques/approaches used (e.g. diversified, Gonstead, BCP), and professional membership status.

#### Consent

A consent form will be completed by chiropractors (Additional file 3). Participating chiropractors will explain the study to patients and obtain informed consent from interested patients (Additional file 4). We do not expect that study participation will cause any harm to participants. If, at any time, participants decide they would like to withdraw, they will be able do so without any consequences to their routine management.

#### Randomization methods (generation of a random sequence)

Chiropractors within recruited practices meeting the inclusion criteria will be randomly allocated to receive either the KT strategies plus practice guidelines for the intervention group or practice guideline alone for the control group. Randomization will be done in a 1:1 ratio to the intervention and control groups using Stat Trek’s Random Number Generator. A research assistant independent of the study will generate, implement, and protect the randomization sequence.

#### Concealment of the allocation sequence

The allocation procedure will be conducted by a researcher using http://stattrek.com/statistics/random-number-generator.aspx. We will provide the ID numbers representing each recruited participant, and he/she will inform us whether the participant is allocated to the intervention or control group. Results will be communicated to the study coordinating unit. Thus, strict separation will be maintained between the code sequence generator and the study coordination personnel.

#### Blinding

Investigators not involved in the delivery of the intervention and the study statistician will be blinded to group allocation until the statistical analysis has been completed. Participating chiropractors will be aware of the KT interventions they are receiving/implementing; otherwise, they will not be explicitly informed of their assigned groups. Participating chiropractors will be instructed not to tell their patients about their KT interventions so as to maintain patient blinding. Participating chiropractors will also be kept blind to all study hypotheses.

### Data collection and management

#### Retention

Follow-up, in terms of both attended appointments and completion of questionnaires, will be assessed at the end of the study.

#### Logistics of multicentre procedures

Challenges in recruiting patients across practices will highlight possible difficulties with implementing a multicentre trial. To increase the likelihood of successful recruitment, participating chiropractors will be encouraged to appoint a single staff member who will be responsible for local trial operationalization and recruitment.

### Statistical analysis

The main analysis will focus on descriptive statistics relating to feasibility to estimate likely recruitment and retention rates, adherence to the intervention, and provide key parameters, e.g. effect size needed to decide on a primary outcome and to estimate the sample size for a full-scale study. Also, we will estimate the potential efficacy of the intervention on adherence to the recommended multimodal approach for NSNP and on patient outcomes of pain and disability. Instead of calculating an average response on each measure for each group and comparing means between groups, this study will identify the proportion of people in each group making a treatment response, which will then be compared between groups. Each person will be classified as having made a response, a deterioration, or no change on each measure based on a change equal to or greater than the MCID published or recommended for that measure. Table 4 provides the summary of this analysis.

**Table 4:** Statistical analysis

Construct	Measure	Measurement scale
Feasibility of recruitment, retention, and data completion (both chiropractors and patients)	Recruitment and retention rates, missing data	Descriptive statistics: mean and SD for continuous variables and proportions for categorical variables
Chiropractor process outcomes		
Adherence rate	Single indicators	Continuous data (number of DCs adhering to the intervention per number of eligible participants)
	Composite	Count of indicators reaching “success” threshold
Behavioural	Level of knowledge and self-efficacy	5-point Likert scale (from 1 = strongly agree to 5 = strongly disagree), ordinal
Patient health outcomes		
Pain	Visual analogue scale	Self-rated level of pain on 11 points, continuous
Disability	Neck Disability Index	Scale range and subscales: 10 items in total; each item is scored from 0 to 5 (“0” = no disability and “5” = full disability) for a total of 50
Satisfaction with care	Questionnaire	Self-rated satisfaction with care measured on the short-form questionnaire rated on a 5-point Likert scale from “very satisfied” to very “dissatisfied”
Potential efficacy	Post-intervention between groups	Normal approximation to the binomial

For the efficacy potential analysis, we will estimate the proportion of chiropractors who endorsed a higher knowledge level post-intervention in the control group and use this as the basis for calculating the probability of achieving a response more extreme than this in the intervention group using the normal approximation to the binomial distribution (stattrek.com). For example, if 2 of 15 participants in the control group endorse a higher knowledge level, this yields an expected “success” proportion of 0.13. Based on this expected proportion or probability, if 5 or more of the 16 chiropractors in the intervention group endorsed a higher knowledge response, the probability of this occurring by chance would be 0.047. This approach will be used for each of the single indicator variables under study here (i.e. knowledge and self-efficacy).

We will use generalized estimating equations (GEEs) to adjust for anticipated correlation in measurements of observations within practices (clusters). For pain and disability, GEE will be used separately for each of these outcomes and to examine for any effect of our complex intervention over time (the follow-up period). This method extends the standard regression analysis to account for any covariance between repeated measurements of pain and disability (as separate outcomes) over time, in addition to accounting for anticipated correlation of observations within practices.

#### Missing data

Missing data will be assumed not to be at random, especially if there are differential losses to follow-up between groups. However, no imputation of missing data is planned.

#### Dissemination of results

**Fig. 1:**
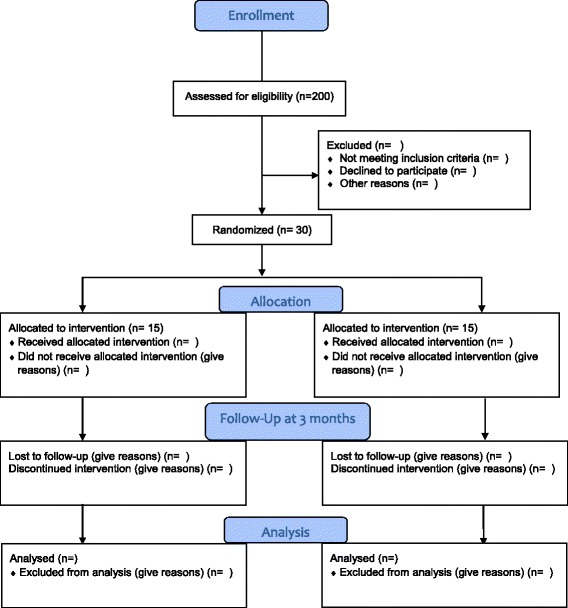
CONSORT participant flow diagram

The results of the study will be published in a peer-reviewed journal and presented at the CCA National Convention. The study will be reported using the Consolidated Standards of Reporting Trials (CONSORT) diagram (Fig. 1). A summary of the study results will also be saved at ClinicalTrials.gov to allow general access to obtain findings.

## Discussion

We propose to determine the feasibility of evaluating a KT intervention in chiropractic clinical practice designed to improve the management of NSNP. The primary purposes of a feasibility study are to ensure that study implementation is practical and to reduce threats to the validity of a larger fully powered study [43]. This study is primarily a feasibility study with feasibility objectives. As a “small-scale” version of a planned main study, this study also constitutes a pilot study aimed at testing whether the components of the main study can all work together.

A full-scale randomized controlled trial in Canada would aim to determine whether the use of multimodal care in patients with NSNP reduces the pain and cost of treatment. A confirmatory trial should provide key insights on the effects and advantages of the use of multimodal care by chiropractors on patient health outcomes (e.g. pain, physical functioning, disability, and satisfaction with care) and inform health service-related research questions and interventions that could be translated into existing health care systems. A better understanding of the effective components of multimodal care, and the effective implementation of CPGs in general, should help future researchers and chiropractors to design and implement complex KT interventions and multimodal care aimed at maximizing the uptake and utilization of evidence in the management of patients with NSNP.

### Study limitations

Given the nature of the study, we will be unable to determine which individual components of the intervention will be effective or ineffective or to quantify the number of modalities administered to individual patients. Recruitment is always a concern in clinical studies, and we anticipate difficulty with participation from busy, community-based private chiropractors. Hence, we are aware that it is important to establish the feasibility of conducting the current study within private chiropractic practices in Canada before progressing to the full-scale study. Meeting this objective is necessary for the purposes of future funding applications for our planned main study.

### Relevance to practice

We are not aware of published studies on the successes and failures of previous attempts to implement a multifaceted KT strategy aimed at improving the management of NSNP by chiropractors. The conduct of this feasibility study is expected to be compatible with existing infrastructure while permitting a certain degree of flexibility and adaptation to the needs and routines of individual community-based clinicians. In the current pilot portion of the study, we will test chiropractors’ adherence to (and therefore tolerance of) a study protocol aiming to both confirm the effectiveness and increase the use of multimodal care (a combination of manual therapy, self-management advice and support to patients, and promotion of physical activity including exercise) for patients with NSNP in a future main study.

Our future main study will also provide insight into the effect of multimodal care on physical functioning, quality of life, and other outcomes important for patient and provider decision-making. It will serve as a template for additional trials of knowledge implementation and complex evidence-based chiropractic intervention studies. While the proximate goal is to improve chiropractors’ receptiveness to and utilization of research evidence in private practice, the ultimate goal is to optimize the outcomes of patients managed by chiropractors, both for NSNP specifically and for other musculoskeletal conditions in general in the community.

### Trial status

At the time of writing, 45 chiropractors have agreed to participate in the study and have been randomized to the intervention and control groups. Participants have started recruiting neck pain patients. Results are expected in May 2016.

## Abbreviations

BAP, Brief Action Planning; CCA, Canadian Chiropractic Association; CPGs, clinical practice guidelines; KT, knowledge translation; NDI, Neck Disability Index; NSNP, non-specific neck pain; PSQ-18, Patient Satisfaction Questionnaire; TDF, Theoretical Domains Framework; VAS, visual analogue scale

## Additional files

Additional file 1: Brief Action Planning skill survey. (PDF 228 kb)
Additional file 2: Specific objectives of the intervention components. (PDF 180 kb)
Additional file 3: Chiropractor information sheet and consent form. (PDF 245 kb)
Additional file 4: Patient information sheet and consent form. (PDF 312 kb)
