# Supplementary material for: Testing the feasibility of a knowledge translation intervention designed to improve chiropractic care for adults with neck pain disorders: study protocol for a pilot cluster-randomized controlled trial
Source: Pilot Feasibility Stud. 2016 Jul 20;2:33. doi: 10.1186/s40814-016-0076-9 (PMC5154031; doi:10.1186/s40814-016-0076-9)
Supplement: Additional file 1: — Brief Action Planning skill survey. (PDF 228 kb) [file 40814_2016_76_MOESM1_ESM.pdf]

Additional file1

<u>Brief Action Planning skills survey</u>

Please check the box with ✓ that best describes the skills covered in any training you have had (including the recent BAP training) and your experiences with those skills. The responses range from left to right, starting with not having an opportunity to learn about it, to being confident using a skill you have learned. You may choose one answer per row.

	**I don't know much about this**	**I tried to practice this during my training**	**I use this skill in my work**	**I am confident using this skill routinely in my work**
The spirit of Motivational Interviewing				
Teach-back for health literacy				
Using reflections to emphasize hope and encourage change				
Helping patients create action plans				
Collaborative problem solving				
Checking in on action plans				
Using a confidence scale or ruler				
Helping patients to talk about change				
Developing strategies for working with challenging situations and people				
Using Ask-Tell-Ask when giving information or advice				

Brief Action Planning (BAP)

Pre and Post-intervention questionnaire

This questionnaire was design to help us gather more information about your experience of using the BAP tool in your practice.

Read carefully the following statements. For each statement, please answer by circling ONE number which best applies to you.

Let's Be Better Health Care Partners: We Want *Your* Feedback!

1. **How important is it for you to explain things in a way your patients can understand?**

0 1 2 3 4 5 6 7 8 9 10

Not at all important Extremely important

- **How confident are you that you can explain things in a way that your patients can understand?**

0 1 2 3 4 5 6 7 8 9 10

Not at all confident Extremely confident

2. **How important is it for you to elicit your patients' preferences and cultural traditions when planning their care?**

0 1 2 3 4 5 6 7 8 9 10

Not at all important Extremely important

- **How confident are you that you can elicit your patients' preferences and cultural traditions when planning their care?**

0 1 2 3 4 5 6 7 8 9 10

Not at all confident Extremely confident

3. **How important is it for you to work *collaboratively* with your patients to set goals to help them to improve their health and well-being?**

0 1 2 3 4 5 6 7 8 9 10

Not important Very important

- **How confident are you that you can work *collaboratively* with your patients to set goals to help them to improve their health and well-being?**

0 1 2 3 4 5 6 7 8 9 10

Not at all confident Extremely confident

4. **How important is it for you to address the barriers that your patients' face in setting or reaching their goals?**

0 1 2 3 4 5 6 7 8 9 10

Not at all important Extremely important

- **How confident are you that you can address the barriers that your patients' face in setting or reaching their goals?**

0 1 2 3 4 5 6 7 8 9 10

Not at all confident Extremely confident

5. **How important is it for you to arrange or provide follow up with your patients regarding their goals?**

0 1 2 3 4 5 6 7 8 9 10

Not at all important Extremely important

- **How confident are you that you can arrange or provide follow up with your patients regarding their goals?**

0 1 2 3 4 5 6 7 8 9 10

Not at all confident Extremely confident

6. **How important is it for you to talk with your patients about including family or other supporters in a care plan?**

0 1 2 3 4 5 6 7 8 9 10

Not at all important Extremely important

- **How confident are you that you can talk with your patients about including family or other supporters in a care plan?**

0 1 2 3 4 5 6 7 8 9 10

Not at all confident Extremely confident

7. **How important is it for you to work with your patients to facilitate behavior changes that will make their health better?**

0 1 2 3 4 5 6 7 8 9 10

Not at all important Extremely important

- **How confident are you that you can work with your patients to facilitate behavior changes that will make their health better?**

0 1 2 3 4 5 6 7 8 9 10

Not at all confident Extremely confident

Patient Survey - Brief Action Planning

Put a *X* in the box that best describes how confident you are:

	**Not Very Confident** 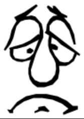	**Somewhat Confident** 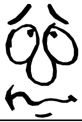	**Very Confident** 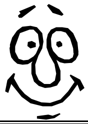	**I do not have any health problems**
How confident (sure) are you that you can control and manage most of your health problems?				

copyright FNX Corp. (USA) and the Trustees for Dartmouth College

**Support for Healthy Behaviors**					
Staying healthy can be challenging. We would like to learn about the type of help you get from your chiropractor. Your answers will be kept confidential and will not be shared with your chiropractor.					
**Over the past 3 months, when I received health care, I was:**					
	None of the time	A Little of the Time	Some of the Time	Most of the Time	Always
1. Asked to talk about my goals for my health.	☐$_1$	☐$_2$	☐$_3$	☐$_4$	☐$_5$
2. Asked for my ideas when we made a plan for my health.	☐$_1$	☐$_2$	☐$_3$	☐$_4$	☐$_5$
3. Helped to set specific plans to improve my health, such as eating or exercise.	☐$_1$	☐$_2$	☐$_3$	☐$_4$	☐$_5$
4. Helped to make a plan that I could carry out in my daily life.	☐$_1$	☐$_2$	☐$_3$	☐$_4$	☐$_5$
5. Contacted me after a visit to see how things were going.	☐$_1$	☐$_2$	☐$_3$	☐$_4$	☐$_5$
