# Supplementary material for: Testing the feasibility of a knowledge translation intervention designed to improve chiropractic care for adults with neck pain disorders: study protocol for a pilot cluster-randomized controlled trial
Source: Pilot Feasibility Stud. 2016 Jul 20;2:33. doi: 10.1186/s40814-016-0076-9 (PMC5154031; doi:10.1186/s40814-016-0076-9)
Supplement: Additional file 2: — Specific objectives of the intervention components. (PDF 180 kb) [file 40814_2016_76_MOESM2_ESM.pdf]

Additional file2

Specific objectives of the intervention components

A. Webinar series:

Webinar 1: Evidence-Informed Practice & Clinical Practice Guidelines

- Understand the importance of 'Evidence-Informed Practice'
- Report barriers and potential solutions to applying 'Evidence-Informed Practice' in day-to-day practice
- Discuss the characteristics, strengths and limitations of 'Clinical Practice Guidelines'
- Review the evolution of the Canadian Chiropractic Guideline Initiative and recognize its implications for chiropractors, patients and the chiropractic profession
- Review the methodological approach utilized to create the new Neck Pain Guidelines
- Understand the conditions the Neck Pain Guidelines apply to familiarize yourself with resources for staying up to date, including the new Canadian Chiropractic Guideline Initiative website.

Webinar 2: Neck Pain: Clinical Practice Guidelines

- Describe the diagnostic triage necessary for determining high risk and low risk patients and determining appropriate strategies for care;
- Describe key findings of the Neck Pain Guidelines and their implications for clinical practice;
- Describe the advantages of multimodal care;
- Complete two clinical vignettes designed to assist clinicians in applying specific Neck Pain Guideline recommendations for imaging and multimodal care.

Webinar 3: Simple ways to effectively implement self-management strategies

- Learn the process and the attitudes of self-management strategies;
- Become familiar with the process of Brief Action Planning;
- Become familiar with the attitudes to adopt when using it with a patient;

B. Video on the BAP: (15-minute learning module with segmented video to help apply material learned on self-management and Brief Action Planning).

- Become familiar with the process of Brief Action Planning.
- Become familiar with the attitudes to adopt when using it with a patient.
