# Supplementary material for: Testing the feasibility of a knowledge translation intervention designed to improve chiropractic care for adults with neck pain disorders: study protocol for a pilot cluster-randomized controlled trial
Source: Pilot Feasibility Stud. 2016 Jul 20;2:33. doi: 10.1186/s40814-016-0076-9 (PMC5154031; doi:10.1186/s40814-016-0076-9)
Supplement: Additional file 3: — Chiropractor information sheet and consent form. (PDF 245 kb) [file 40814_2016_76_MOESM3_ESM.pdf]

Additional file 3
Chiropractors Information Sheet and Consent Form

"Testing the feasibility of a knowledge translation intervention designed to improve chiropractic care for adults with neck pain disorders"

Principal study Investigator:
Dr. André Bussières, DC, PhD, Assistant Professor, School of Physical and Occupational Therapy, Faculty of Medicine, McGill University

1. **Introduction:**

You are being asked to participate in this research project because as a licensed chiropractor you regularly manage patients with neck pain. Please read this 'Information Sheet and Consent Form' carefully and ask as many questions as you like before deciding whether to participate in this research study. You can discuss this decision with your colleagues.

Participation in this study is voluntary.You arefree torefuse to participate or to withdraw your consent to participate at any time.Refusing to participate will involve no penalty or loss of benefits. There is no penalty for withdrawing and all your collected data will be destroyed.

2. Background, Purpose and Design of the Study:

Current evidence suggests a multimodal approach including manual therapy, advice about self-management, and physical activity including exercise is an effective treatment strategy for acute and chronic neck pain. However, research shows that the transfer of evidence into practice is unpredictable and can be a slow and haphazard process. The purpose of this pilot project is to determine the feasibility of implementing a knowledge translation intervention designed to improve the management of non-specific neck pain. We plan to build on the results of this feasibility study by formally evaluating the strategies to integrate evidence-based multimodal care approach into clinical practice for individuals with nonspecific neck pain and methods for bringing new research findings into practice. Also, this study will help us determine the best way to measure the effects of treatment. The study design is a feasibility study of chiropractors who will receive a KT intervention. We anticipate 40 licensed chiropractors to take part in the study.

3. Study Procedures:

If you agree to take part in this study, you will be randomly assigned to receive either the KT intervention plus a copy of the clinical practice guidelines neck pain management for the intervention group, or the clinical practice guideline alone for the control group. The randomization is 1:1. You will be asked to complete a demographic questionnaire that consists of your name, age, gender, chiropractic school attended, area of practice, number of patients you treat daily and techniques used. It should be completed after signing the consent form.

Practitioners randomized to the intervention group will be asked to watch three webinars on the neck pain guideline, each of a length of 50-60 minutes, a learning module on self-management of 22 minutes in length, and two clinical vignettes, each taking about 15-20 minutes to complete. These activities can be complete at your own pace preferably within the same week. You will be asked to implement the KT strategies you have learned on patients with acute or chronic neck pain you have recruited for the study. We anticipate that only about 45minutes are needed to complete the study forms for each recruited patient.

4. Possible Side Effects and/or Risks:
There are no known risks to chiropractors and patients.

5. Benefits of the Study:
Your participation in this research may allow the researchers to gain in depth understanding of how best to transfer new knowledge strategies to chiropractors. This in turn may be of benefit to future patients.

6. Confidentiality:
No identifying information will be reported in any publications, reports or presentations. Confidentiality of the data will be protected by assigning each participant such as yourself a unique identification number replacing the name and the registration number of care providers and using that number on all data about participation. The data will be use to evaluate the feasibility to implement the KT intervention within chiropractic practice. All paper records will be stored in a locked office. Only the principal investigator of the studywill access your data. All electronic records will be stored at the administrative Services Building of McGill University and protected by a user password. The study data retention is for 7 years after which time the data will be destroyed.

7. Compensation:
If you agree to participate in the study, you will be entered into a draw to win one of four $250 gift certificates from Amazon as a token of appreciation. Please note if you were randomized to the usual care group, the webinars and online module may be watched at any time after the completion of the study by registering on CE Connect system on the Canadian Memorial Chiropractic College (CMCC) website.Most provincial chiropractic regulatory boards have pre-approved the KT intervention for four hours of Continuing Education (CE). Certificates of completion are produced once the KT intervention is completed and all quizzes have been successfully answered. Certificates must be submitted to your regulatory board so you are eligible to receive the CE credit hours. Please note that recognition of CE activities can vary from one province to another. Please communicate with the Registrar in your province if you have questions about the process.

8. Questions about the Study:
A. If you have any questions or concerns, please contact Dr. André Bussières at andre.bussieres@mcgill.ca. or by phone: (514) 398-4400 ext. 00849

B. If you have any questions about your rights as a research participant, please contact IldeLepore, McGill IRB Ethics Officer, by email: ilde.lepore@mcgill.ca or by phone: 514-398-8302

9. Returning the signed consent form and demographic questionnaire:
Please mail it back to our office in the pre-paid envelope.

The McGill University Institutional Review Board (IRB) has reviewed this study. The IRB considers the ethical aspects of all research studies involving human subjects at McGill University.

<u>CONSENT FORM</u>

"Testing the feasibility of a knowledge translation intervention designed to improve chiropractic care for adults with neck pain disorders"

Consent to Participate in Research

I am aware that I am being asked to participate in a research study that seeks to assess the feasibility of implementing a knowledge translation intervention designed to improve the management of non-specific neck pain.

I have read this consent form. I have been informed of the purpose of this study, and I am aware of the study procedures, and the risks and benefits of taking part. I have asked any questions I had, and my questions were answered. I have been informed that participation in this study is voluntary, and that I can withdraw from this study at any time without giving a reason. I agree to take part in this research study. I do not give up any of my legal rights by signing this consent form.

Signatures

____
Participant's Name (PleasePrint)

______________________________ ________________
Participant's Signature Date

____
Name of Investigator/Delegate (Please Print)

____________________________________ ________________
Signature Date
