# Supplementary material for: Testing the feasibility of a knowledge translation intervention designed to improve chiropractic care for adults with neck pain disorders: study protocol for a pilot cluster-randomized controlled trial
Source: Pilot Feasibility Stud. 2016 Jul 20;2:33. doi: 10.1186/s40814-016-0076-9 (PMC5154031; doi:10.1186/s40814-016-0076-9)
Supplement: Additional file 4: — Patient information sheet and consent form. (PDF 312 kb) [file 40814_2016_76_MOESM4_ESM.pdf]

Additional file 4
Patient Information Sheet and Consent Form

“Testing the feasibility of a knowledge translation intervention designed to improve chiropractic care for adults with neck pain disorders”

Principal Study Investigator:
Dr. André Bussières, DC, PhD, Assistant Professor, School of Physical and Occupational Therapy, Faculty of Medicine, McGill University.

1. INTRODUCTION:
You are invited to participate in a research study conducted by McGill University and the Canadian Chiropractic Guideline Initiative (CCGI) because you have neck pain and are undergoing care. The research team has developed a strategy to increase the use of multimodal care by chiropractors who are treating patients with neck pain. We are enrolling patients with neck pain across chiropractic practices in Canada.

In order to decide whether or not you want to be a part of this research study, you should be aware what is involved in the study and the potential risks and benefits. This form gives detailed information about the research study, which will be discussed with you. Once you understand the study, you will be asked to sign this form if you wish to participate. Please take your time to make your decision.

If you volunteer to be in this study, you may withdraw at any time. This will in no way affect the quality of care you receive at this clinic. You may also refuse to answer any questions that you do not want to answer and still remain in the study. The investigator may withdraw you from this research if circumstances arise which warrant doing so.

2. WHY IS THIS RESEARCH BEING DONE?
The objective of our research is to provide a foundation for understanding clinical responses to multimodal care in patients with non-specific neck pain. It involves testing a strategy for clinicians to increase the use of multimodal care and using clinically relevant and feasible outcome measures. Multimodal care means the use of two or more treatment modalities,including spinal manipulative therapy, mobilization, massage, exercise, and advice on self-management.

We plan to build on the results of this feasibility study by evaluating the strategies to integrate an evidence-based multimodal care approach into clinical practice for individuals with nonspecific neck pain.

3. STUDY PROCEDURES?
The researchers are comparing two methods of treatment currently used and approved in clinical care. You will be asked to attend your regular chiropractic visits for the treatment of your neck pain condition. Each visit may last between 10-20 minutes. Depending on the severity of your

condition and how your neck pain responds to the treatments, your chiropractor may recommendyou receive 1 to 3 visits per week for a duration of 2-6 weeks. The treatment plan provided by your chiropractor will remain essentially the same throughout the procedure. You will be asked to complete questionnairesto help us assess your pain and function levels.

4. WHAT ARE THE POSSIBLE RISKS?
There are no major risks to the safety of the patients in this study.

5. WHAT ARE THE POSSIBLE BENEFITS FOR ME AND/OR FOR SOCIETY?
We cannot promise any personal benefits to you from your participation in this study. By participating in this study, you will help healthcare workers better understand how to treat non-specific neck pain and determine the best dosage of multimodal care approach.

6. IF I DO NOT WANT TO TAKE PART IN THE STUDY, ARE THERE OTHER CHOICES?
It is important for you to know that you can choose not to take part in the study. If you do not wish to participate, we respect your decision and it will in no way affect your care or treatment and you may continue to receive your regular chiropractic care.

7. CONFIDENTIALITY?
No identifying information will be reported in any publications, reports or presentations. Confidentiality of the data will be protected by assigning each participant such as yourself a unique identification number replacing the name and the registration number of care providers and using that number on all data about participation. All paper records will be stored in a locked office. Only the principal investigator will have access to your data. All electronic records will be stored at the administrative services building of McGill University and protected by a user password. The study data retention is for 7 years after which time the data will be destroyed. For the research purposes, organizations involved in the study may audit your records.

9. WILL I BE COMPENSATE TO PARTICIPATE IN THIS STUDY?
You will not receive any reimbursements for any costs (e.g. travel or parking) for taking part in this study**.**

10. WILL THERE BE ANY COSTS?
Your participation in this research project does not involve additional costs to you.

11. IF I HAVE ANY QUESTIONS OR PROBLEMS, WHOM CAN I CALL?
A. If you have any questions or concerns about the study, please contact Dr. André Bussières at andre.bussieres@mcgill.ca. or by phone: 514-398-4400 ext-00489.

B. If you have any questions about your rights as a research participant, please contact IldeLepore, McGill IRB Ethics Officer, by email: ilde.lepore@mcgill.ca or by phone: 514-398-8302

CONSENT STATEMENT

"Testing the feasibility of a knowledge translation intervention designed to improve chiropractic care for adults with neck pain disorders"

SIGNATURE OF RESEARCH PARTICIPANT

I have read this consent form. I have been informed of the purpose of this study, and I am aware of the study procedures, and the risks and benefits of taking part. I have asked any questions I had, and my questions were answered. I have been informed that participation in this study is voluntary, and that I can withdraw from this study at any time without giving a reason. I agree to take part in this research study. I will receive a signed and dated copy of this consent form. I do not give up any of my legal rights by signing this consent form.

____

Name of Participant

____

Signature of ParticipantDate

Consent form administered and explained in person by:

I acknowledge the receipt of participant's consent form and my responsibility for the care and well-being of the above research participant, to respect the rights and wishes of the research participant, and to conduct the study according to applicable Good Clinical Practice guidelines and regulations.

Principal Investigator of Study:

____

Name and title

____________________________________ ______________

Signature Date
